# Information propagation within the Genetic Network of Saccharomyces cerevisiae

**DOI:** 10.1186/1752-0509-4-143

**Published:** 2010-10-26

**Authors:** Sharif Chowdhury, Jason Lloyd-Price, Olli-Pekka Smolander, Wayne CV Baici, Timothy R Hughes, Olli Yli-Harja, Gordon Chua, Andre S Ribeiro

**Affiliations:** 1 Laboratory of Biosystem Dynamics, Computational Systems Biology Research Group, Tampere University of Technology, Tampere, Finland; 2 Banting and Best Department of Medical Research University of Toronto, 160 College St. Room 1302. Toronto, ON, M5 S 3E1 Canada; 3 Institute for Systems Biology, 1441N 34th St, Seattle, WA, 98103-8904, USA; 4 Institute for Biocomplexity and Informatics, University of Calgary, Alberta T2N 1N4, Canada; 5 Department of Biological Sciences, University of Calgary, Alberta T2N 1N4, Canada

## Abstract

**Background:**

A gene network's capacity to process information, so as to bind past events to future actions, depends on its structure and logic. From previous and new microarray measurements in *Saccharomyces cerevisiae *following gene deletions and overexpressions, we identify a core gene regulatory network (GRN) of functional interactions between 328 genes and the transfer functions of each gene. Inferred connections are verified by gene enrichment.

**Results:**

We find that this core network has a generalized clustering coefficient that is much higher than chance. The inferred Boolean transfer functions have a mean p-bias of 0.41, and thus similar amounts of activation and repression interactions. However, the distribution of p-biases differs significantly from what is expected by chance that, along with the high mean connectivity, is found to cause the core GRN of *S. cerevisiae*'s to have an overall sensitivity similar to critical Boolean networks. In agreement, we find that the amount of information propagated between nodes in finite time series is much higher in the inferred core GRN of *S. cerevisiae *than what is expected by chance.

**Conclusions:**

We suggest that *S. cerevisiae *is likely to have evolved a core GRN with enhanced information propagation among its genes.

## Background

No general laws have yet been established for how natural selection shapes the large scale topology and logic of gene regulatory networks (GRN). One possible principle shaping the topology of GRNs is that the execution of several internal cellular processes, as well as the proper response to certain external signals, requires specific temporal patterns of expression of multiple genes. To robustly orchestrate a wide spectrum of such complex temporal expression patterns, genes need to constantly exchange information between them.

Within a cell, there are several mechanisms through which genes exchange information. Some are direct, such as interactions via transcription factors (TF), while others are more indirect such as protein-protein interactions [1-3]. Dynamically, GRNs are stochastic [4]. Whether a fluctuation of a protein's level is purely noise or contains information is likely to be context dependent. Most studies assume the dichotomy where a gene's expression level is either "high" or "low". In this view, GRNs are assumed to be binary information processing systems and can be, to some extent, modeled by Boolean networks [5]. Such models aim to capture, at least partially, the information exchange between genes.

In Random Boolean Network (RBN) models of GRNs, nodes represent genes and can have two states: '1' if expressing and '0' otherwise. Nodes update their state synchronously according to Boolean functions of the states of the input nodes. Information propagation in RBNs depends on the network's dynamical regime [6], which can be ordered or chaotic, separated by a phase transition, dubbed "critical" [5].

The dynamical regime of RBN is determined, in general, by its sensitivity, which in turn is determined by its mean connectivity (mean number of connections per node) and mean p-bias (defined as the probability that the output of the Boolean transfer function is '1' for any set of input states) [7].

Relevantly, chaotic RBNs tend to respond widely differently to very similar inputs [8]. In a biological setting, it would not be advantageous for an organism to have a chaotic GRN, given that in common environmental settings, similar inputs require similar responses. On the other hand, ordered networks respond identically to very distinct input signals [8], which in most situations would be disadvantageous.

For that reason, near-critical gene networks are likely to be naturally favored. If so, this imposes constraints in the topology and logic of evolved gene networks, namely on its sensitivity.

Several studies have evaluated information propagation in RBNs. One measure that has been used is the basin entropy, which characterizes the number and size of the basins of attraction and hence the ability to respond differently to different inputs [9]. Another such measure is the mutual information between subsequent states of single nodes [10]. Both quantities were found to be maximum in critical RBNs [9,10]. A study of information propagation in Boolean networks where all nodes are driven by a common input signal found that critical RBNs best distinguished differences between inputs states and were able to perform the most complex computations on time series [11]. In [12], it was observed that ensembles of critical RBNs have broader distributions of dynamical behaviors.

The amount of mutual information contained in the time series of two elements gives a measure of how well their activities are coordinated (in the sense that, given the state of one element, one can, to some extent, predict the other's state). In RBNs, coordination between nodes' states arises from the fact that the future state of a node is determined by the present state of its input nodes. Mean temporal pairwise mutual information (*I*) has therefore been used as an estimate of the quantity of information propagated between nodes within a RBN [6]. For infinite size networks, critical RBNs maximize *I*, while the maximization occurs slightly in the chaotic regime for finite size networks [6].

The ability of critical RBNs to better distinguish different signals and respond similarly to similar signals is expressed in *I*'s maximization [6]. Since the correct execution of cellular functions depends on the GRN's information processing capacity, it is likely that this is under selective pressure. It is therefore of interest to study information propagation in models of GRNs [13].

Here we first infer, from microarray measurements, a functional topology of the GRN of *S. cerevisiae*. Since we focus on direct information propagation between genes, we extract a "core network" of genes interacting directly with one another, each having both inputs and outputs. Gene enrichment methods are used to verify whether the inferred interactions have some parallel to known relationships between genes. Next, we infer the Boolean transfer functions of each gene of this core network. We test the inferred network for self-consistency with the measurements. Finally, we address the following question: Do the topology and logic, both globally and locally, favor information propagation in the core network? I.e., has this core network evolved towards maximizing information propagation and what are its limitations in this regard?

## Methods

We model the GRNs (both the inferred core network as well as the null model networks) using the Boolean network modeling strategy [5], which was found to be able to mimic, to some extent, results from deletion and overexpression measurements in GRNs [14]. This is a very simplistic modeling strategy of GRNs. The dynamics of real GRNs are stochastic, the protein and RNA levels are not binary quantities, and the genes in a real GRN do not change their expression levels synchronously. Nevertheless, when compared with the stochastic modeling strategy [4], generally considered the most accurate one, the Boolean modeling strategy proved itself more realistic regarding propagation of changes in expression levels than common O.D.E. models, among others [15]. Unfortunately, it is computationally unfeasible at the moment to use the stochastic modeling strategy or the delayed stochastic modeling strategy [16] for gene networks of large size and complexity, thus, we opted for the Boolean approach. Since there is little agreement on how to introduce noise in the Boolean approach (such as using random bit flips or asynchronous update schemes), we use the synchronous, noiseless model.

### Microarray Measurements

We infer the topology and logic of the network between 328 genes of *S. cerevisiae *from microarrays from 292 conditional essential mutants (data set 1, from [17]), 40 strains overexpressing a unique transcription factor gene (data set 2, from [18]), and 84 new perturbation experiments (data set 3). Data set 3 is provided in Additional file 1. Microarray measurements were performed as described in [18,19]. In all cases, the expression levels are compared to wild type.

### Inference of the topology of the core gene network of S. cerevisiae

From the Yeastract http://www.yeastract.com list of binding interactions in the *S. cerevisiae *GRN, we estimate the mean connectivity (*K*) among the 328 genes to be 5.6. Note that these interactions are not all necessarily functional. This mean *K *is only used to determine a reasonable threshold on the minimum effect a gene must have on another gene's expression level when it is deleted or overexpressed. The threshold that best fits this requirement is a 3.32-fold change in expression.

Next, we extract a subnetwork of the inferred network, including only genes that can have both inputs and outputs, as only these can receive and propagate information to and from other genes. From here onwards, we study the structure and dynamics of this inferred "core network" (input matrix in Additional file 2).

### Inference of the Boolean Transfer Function of each gene

Since the measurements only provide information of the output state for some of the possible inputs states, and each gene usually has multiple inputs, we set up rules in order to infer complete transfer functions to be able to simulate the inferred network's dynamics. These rules are implemented in an algorithm that goes as follows:

1. Determine the degree of change in the expression of a gene, given the deletion or overexpression of another gene. If the degree of change is 3.32-fold this gene is assumed to be a direct input of the other gene (see previous section).

Let gene *G *have *n *input genes: *i*_1_, ..., *i_n_*. Let the expression level of gene *G *when when input *i_j _*is overexpressed or deleted be denoted as *E*(*G*, *j*, *x*), where *x *denotes overexpression or deletion, while its wild type expression is denoted as *E_WT _*(*G*). We define the "weight" of gene *i_j _*on gene *G *as:

$$W\left(G,j,x\right)=\text{sign}\left(\ln\left(\frac{E(G,j,x)}{E_{WT}\left(G\right)}\right)\right)\cdot \max\left(\frac{E(G,j,x)}{E_{WT}\left(G\right)},\frac{E_{WT}\left(G\right)}{E(G,j,x)}\right)$$

In this expression, sign is the sign function, and max is the "max" function that returns the maximum number of a list of values. When no data exists for a particular *j *and *x*, *W *(*G*, *j*, *x*) is defined to be 0.

2. For a given state of the input genes ***x ***= *x*_1_, ..., *x_n_*, we calculate the expected expression level of the output gene by the sum of the weights from the overexpression and deletion experiments corresponding to the input states:

$$V\left(G,x\right)=\sum_{j=1}^{n}W\left(G,j,x_{j}\right)$$

3. If *V *(*G*, ***x ***is positive, then the corresponding entry in the truth table is set to 1, while if it is negative it is set to 0. If the sum is 0, the corresponding entry is randomly chosen.

The fraction of values in the truth tables that must be set randomly is always at most 2^-*n *^per gene, where *n *is number of inputs. For the experimental data used here to infer the core network, for each gene, less than 2% of its output states were set randomly, since there is always at least one measurement of expressions levels after deletion or overexpression for each input of each gene in the core network (given its definition).

### Connectivity, p-bias, clustering coefficient, path length, sensitivity and mutual information

Having inferred the topology and transfer functions of the core network of *S. cerevisiae*, we now compute several topological features such as the mean p-bias, defined as the mean over all nodes of the fraction of inputs states which cause the output state in the next time moment to be equal to 1. As we study the ability of the GRN to propagate information, we also calculate the mean directed path length *L*, the generalized clustering coefficient, *C_p_*, and the mean sensitivity, *S*, as these quantities (defined below) are known to affect information propagation between the nodes in an RBN [6].

The mean directed path length, *L*, of an RBN is obtained by computing the path length between each pair of nodes with a direct path between them, and averaging over the number of such pairs. Pairs of nodes without a path between them do not contribute to the mean *L*. The value of *L *can thus be somewhat deceptive, as one can have a network with disconnected clusters with lower *L *than a network where all nodes belong to the same cluster (a cluster being a set of nodes such that there is an undirected path between all pairs of nodes). For this reason, we also report the number of "disconnected clusters".

The clustering coefficient *C*, as originally defined [20], measures the fraction of effective connections between the first nearest neighbors of a node in an undirected graph, out of the total number of possible connections. Let *E_i _*be the number of connections between the *k_i _*nodes connected to a node *i*, in a network with a total of *N *nodes. The average *C *of the network is:

$$C=\frac{1}{N}\sum_{i=1}^{N}\frac{2E_{i}}{k_{i}\left(k_{i}-1\right)}$$

Previous work has shown that the *I *of RBNs is highly dependent on other local topological structures besides triangles, such as squares, self-connections, etc [21]. For this reason, the concept of clustering coefficient [20] was extended to *C_p_*, the generalized clustering coefficient, that accounts for any loops containing *p *nodes [21]. Let *i *be the node index, and $\kappa_{r_{1,}r_{2}}^{i}$ be the amount of connections between the nodes at path length distance *r*_1 _and the nodes at distance *r*_2 _from *i*. Let $T_{r_{1,}r_{2}}^{i}$ be the maximum possible number of such connections. *C_p_*, for *p *> 2, is given by [21]:

$$C_{p}=\frac{1}{N}\sum_{i=1}^{N}\left(\frac{\sum_{r=1}^{p-2}\kappa_{r,p-r-1}^{i}}{\sum_{r=1}^{p-2}T_{r,p-r-1}^{i}}\right)$$

This expression only applies when *p *is larger than 2. It is further noted that distances between nodes are always calculated so that they are always strictly positive integers. Meanwhile, we define *C*_1 _as the fraction of nodes with self inputs, and *C*_2 _as the mean fraction of bidirectional connections per node. Therefore, if a node has three connections and one is bidirectional, then its contribution to *C*_2 _is 1/3. Note that the definition of *C*_3 _matches the definition of the original *C *proposed in [20].

*K*, *L *and *C_p _*characterize the topology of the network. To characterize the transfer functions, we calculate their sensitivity. The sensitivity *s^f ^*of a Boolean function *f*, measures how sensitive the output of the function is to changes in the input states [7,22]. The mean sensitivity over all transfer functions in a network (*S*) has been used as an order parameter, that can be used to determine the dynamical regime of the network (order, critical or chaotic) [7], which affects the network's ability to propagate information [21]. The sensitivity *s^f ^*(x) of *f *on input vector x is defined as the number of Hamming neighbors of x on which the function value is different than on x (two vectors are Hamming neighbors if they differ in only one component):

$$s^{f}\left(x\right)=\left|\left\{1\in \left[1,...,\text{k}\right]:f\left(\text{x}⊕e_{l}\right)\neq f\left(\text{x}\right)\right\}\right|=\sum_{l=1}^{k}\chi \left[f\left(\text{x}⊕e_{l}\right)\neq f\left(\text{x}\right)\right]$$

where *e_l _*is the unit vector with 1 in the *l^th ^*position and 0's everywhere else, the ⊕ indicates Exclusive-OR and χ[*A*] is an indicator function that is equal to 1 if and only if *A *is true. The average sensitivity *s^f ^*is given by the expectation of *s^f ^*(x) with respect to the distribution of x [7]. Assuming that the output states of a function are randomly generated following some p-bias *p *independently for each input state, then the average sensitivity of the network can be estimated by: *S *= 2 ×*K *×*p *× (1 *- p*) [7].

While the sensitivity allows us to characterize the dynamical regime of the network, it does not directly inform on the information propagation capability of the network. We use the average pairwise mutual information as a measure of information propagation between the nodes of a RBN. This quantity is defined as in [6]. Let *s_a _*be a process that generates a 0 with probability *p*_0 _and a 1 with probability *p*_1_. The entropy of *s_a _*is [6]:

$$H\left(s_{a}\right)\equiv -p_{0}{\text{ log}}_{\text{2}}\;p_{0}-p_{\text{1}}{\text{ log}}_{\text{2}}\;p_{\text{1}}.$$

Likewise, for a process *s_ab _*generating pairs *xy *with probabilities *p_xy_*, where *x*, *y *∈ {0, 1}, the joint entropy is

$$H\left(s_{ab}\right)\equiv -p_{00}{\text{ log}}_{\text{2}}\;p_{00}-p_{0\text{1}}{\text{ log}}_{\text{2}}\;p_{0\text{1}}-p_{\text{1}0}{\text{ log}}_{\text{2}}\;p_{\text{1}0}-p_{\text{11}}{\text{ log}}_{\text{2}}\;p_{\text{11}}.$$

For a given RBN, we assume infinitely long time series and start from all possible initial states. The fraction of steps for which the value of node *i *is *x *gives *p_x _*for the process *s_i_*. The value of *p_xy _*for the process *s_ij _*is given by the fraction of time steps for which node *i *has the value *x *and *on the next time step *node *j *has the value *y*. Temporal pairwise mutual information between nodes *i *and *j *is then defined as [6]:

$$I_{ij}=H\left(s_{i}\right)+H\left(s_{j}\right)-H\left(s_{ij}\right)$$

where *H*(*s_i_*) is the information-entropy of the time series of states of node *i *at time *t*, *H*(*s_j _*) is the entropy of the time series of states of node *j *at time *t *+ 1, and *H*(*s_ij _*) of the joint state of node *i *at *t *and node *j *at *t *+ 1. With this definition, *I_ij _*measures the extent to which information about the state of node *i *at time *t *influences the state of node *j *one time step later. The propagation may be indirect; a nonzero *I_ij _*may be the result of, for example, the influence of a common ancestor node of both *i *and *j*. Given the definition of *I_ij _*, we use *I*, the mean *I_ij _*for all pairs of nodes, as a measure of information propagation within the network.

### Assessing information propagation and core behavior: null models

To characterize the efficiency of the topology and transfer functions of the inferred core network to propagate information, one has to compare with a null model. We focus on the role of the local structure (*C_p_*) and of the distribution of p-bias. We determine each feature's relevance by comparing with a null model. For that, random networks are generated according to the constraints of the null models and their ability to propagate information is compared with that of the inferred core network of *S. cerevisiae *by computing *I *from time series initialized at a random state.

One null model is used to assess the importance of the degree of *C_p _*of the core. To these null model networks, we impose the same mean *K *as the inferred core network, but connections are placed randomly (for each connection placed, both input and output are randomly chosen from all nodes). We impose a distribution of p-biases in this null model that is identical to the one inferred for the core so that this null model only differs in mean *C_p _*(and thus in the Input and Output distribution). The comparison allows determining whether the observed *C_p _*in the core is likely to have been subject to selection, and if so, what consequences such selection has had on mean *I *.

The other null model is used to assess the effects of the p-bias distribution in the inferred core network of *S. cerevisiae*, as it differs significantly from what is expected by chance. In this null model, we impose the same mean *K*, *C_p _*and p-bias, but the distribution of p-biases is not imposed (how the p-bias of each function is set is described below for both null models).

The topologies of the null-model networks are generated according to the "Random 2" algorithm proposed in [23]. Define *n *as the number of nodes in the graph, and *m *as the number of edges. Given (*k*, *m*) do:

1. Order all node pairs (*u*, *ν*) ∈ [1, *n*]^2 ^in a vector *e*.

2. Set uniformly at random, with probability *n*^-2 ^and without repetition, *m *components of *e *equal to 1.

3. Add an edge from *u *to *ν *if $I_{\left(u,v\right)}$(*e*) = 1.

The imposition of the p-bias distribution in the first null-model (here named "Rand-Beta") was accomplished as follows: for every transfer function, sample a p-bias from the Beta distribution that best fits the inferred core network p-bias distribution, and then generate outputs for that function based on that bias. In the case where the p-bias distribution is "not imposed" (second null model), the p-bias of each function is always 0.41, in agreement with the measured p-bias of the inferred core network (shown in results section). In both cases, once the p-bias of each function from the corresponding distribution, the output for each input state is then randomly set, according to the specific p-bias, independently of all other output states.

For simplicity, we opted to impose only random input-output (I/O) distributions in the null models. A more sophisticated approach that could be taken in the future is to impose the inferred I/O distribution as well (see e.g. [24,25]).

## Results and Discussion

### Topology and Transfer Functions of the inferred Core Network

**Figure 1:**
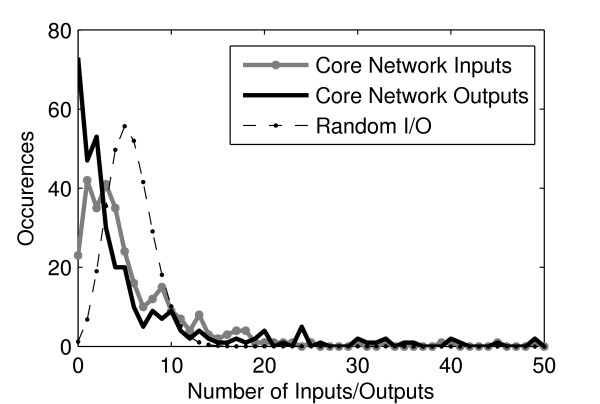
**Input and Output distribution (I/O) of the inferred core network and of the null models**. Input and Output distribution (I/O) of the inferred core network and the I/O distributions of the null models (identical for both null models used here). The null model and inferred core network have same mean *K*.

The inferred core network of *S. cerevisiae *is composed of 328 genes and has a mean connectivity *K *of 5.6. Input and output (I/O) distributions of the inferred core network are shown in Figure 1, which also shows the I/O distributions of networks with the same mean *K*, but random wiring (binomial I/O distributions), for comparison.

Comparing the distributions in Figure 1, *S. cerevisiae *core network exhibits a higher amount of high-degree nodes. Particularly, 8% of the nodes have more than 15 inputs, while in the random networks, this percentage is negligible. Since it is unlikely that heavily combinatorial functions with many inputs can be realized by real genes, it is of interest to analyze the transfer functions of those genes with a high number of inputs. If real GRNs cannot realize highly combinatorial transfer functions, then this input/output distribution would likely force the p-bias distribution to differ significantly from what would be expected by chance. If the p-bias of the transfer functions of individual genes is close to 0.5, they are likely to have complex combinatorial functions. On the other hand, if at the single gene level, the p-bias is biased towards 0 or 1, then highly biased functions are expected (most of input states have the same output state).

**Figure 2:**
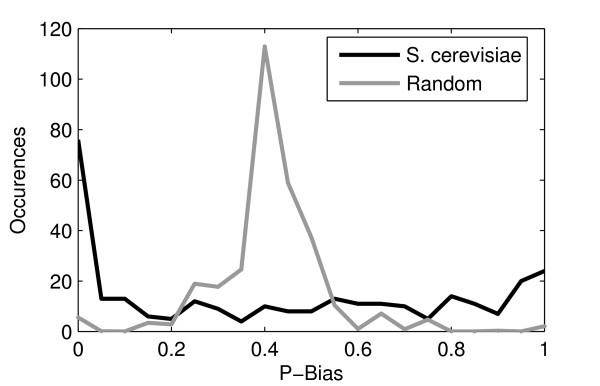
**P-bias distribution of the nodes in the inferred core network and for random p-bias distribution**. P-bias distribution of the nodes in the inferred core network. It looks like a Beta distribution (it is well fitted by a Beta distribution biased to the left with *α *= 0.3467 and *β *= 0.4350). Also shown is a random p-bias distribution for the same mean *K *and p-bias (0.41).

The p-bias distribution of the transfer functions is shown in Figure 2 for *S. cerevisiae *and for randomly generated functions with the same mean p-bias and *K*, for comparison. The p-bias distribution of *S. cerevisiae *is strikingly different from the null model. While the null model has a binomial distribution, *S. cerevisiae*'s is best fit by a Beta distribution with a probability density function *P_f _*(*x*, *α*, *β *) of *α *= 0.3467 and *β *= 0.4350, which is given by:

$$P_{f}\left(x,\alpha ,\beta \right)=\frac{1}{B\left(\alpha ,\beta \right)}x^{\alpha -1}{\left(1-x\right)}^{\beta -1}$$

where *B*(*α*, *β*) is the Beta function that normalizes the total probability to one.

Contrary to what is expected by chance (assuming that all transfer functions can be realized) the transfer functions of the inferred core network of *S. cerevisiae *appear to be highly biased (although unbiased overall). This implies that for most genes with many inputs, almost all their input TFs have similar effects in the expression level (either almost all being repressors or almost all being activators). Relevantly, several eukaryotic genes appear to be predominantly held inactive by chromatin structure [26] (e.g. nucleosomes) and most of its TFs are activators.

Another explanation for the observed p-bias distribution, which does not exclude the first, is that in the genes with multiple inputs, one or two TFs play a dominant role, while the others only have effects in the absence of the dominant ones. Such "dominant" TFs would be expected to have a greater impact on an organism's functioning and cause more severe consequences if their activity is perturbed. Our findings of such TFs, and that they are a minority of all TFs analyzed, agrees with observations in *S. cerevisiae*, where under optimal growth conditions, less that 5% of the TF coding genes are essential (i.e. their deletion causes cell death) [27]. That is, single deletions of most TF genes result in viability under optimal growth conditions, indicating that most TFs are possibly redundant with other TFs. Another alternative, not excluding the first, is that many TFs regulate nonessential processes (or are inactive) under optimal growth conditions [27].

In any case, the shape of the p-bias distribution, resembling a biased "beta-like" distribution with very high variance (hence forth referred to as "beta-like"), suggests that complex combinatorial functions are rare. Relevantly, this is not due to the value of the mean p-bias, as it is 0.41 and thus, not limiting significantly the existence of complex combinatorial transfer functions.

It is interesting to speculate whether the p-bias distribution of *S. cerevisiae *is a consequence of the high mean *K *and the limitations in executing complex transfer functions such as Exclusive-OR, or, if has evolved on its own for a specific purpose (e.g., given *K*, perhaps to cause the network to be near critical). We cannot address this question here, but we can investigate how this distribution affects the mean *I *of the core network. This is done after verifying the degree of accuracy of the inference procedure.

### Verification of the accuracy of inference

The network was inferred based on functional correlations. We therefore expect to find that closely related genes in the inferred core network should have some functional similarity between them, and to be involved in similar biological processes. We test this hypothesis by selecting the output genes of the inferred network with the inputs with which the correlation values are stronger, and then finding functional groups of genes that are enriched in the list of inputs using FunSpec (http://funspec.med.utoronto.ca, as of April 29, 2010). For example, the Transcription Factor Activity and DNA binding gene ontologies are highly enriched in the inferred inputs to HAP4, a global regulator of respiratory gene expression (p-values smaller than 10^-6^). A sample of 10 of the best enrichment scores is shown in Table 1.

**Table 1:** Enriched sets in the list of input genes.

Output Gene	Output Protein Function	Enriched Set	p-value
HAP4	Global regulator of respiratory gene expression	Transcription factor activity	2.723 × 10^-7^
NRG1	Transcriptional repressor	Ribosome biogenesis	1.230 × 10^-6^
CDC19	Pyruvate kinase	Regulation of glycolysis	3.732 × 10^-5^
CDC6	ATP-binding protein required for DNA replication	Ribonuclease MRP activity	6.232 × 10^-5^
UBC9	SUMO-conjugating enzyme	Post-translational protein modification	1.005 × 10^-4^
ORC6	Subunit of the origin recognition complex	DNA replication origin binding	3.362 × 10^-4^
UTP23	Involved in 40 S ribosomal subunit biogenesis	rRNA processing	3.392 × 10^-4^
SEC61	Forms a channel for SRP-dependent protein transport to/from the ER	Enzyme activator	4.117 × 10^-4^
YGR067C	Unknown function	ATPase activator activity	6.005 × 10^-4^
YLR278C	Zinc-cluster protein	Modification with fatty acids	8.531 × 10^-4^

Enriched sets in the list of input genes for selected output genes in the inferred network. Also listed is the cellular function of the protein encoded by the output gene. P-values are calculated with the hypergeometric test. Enrichment was performed by FunSpec (http://funspec.med.utoronto.ca, as of April 29, 2010).

From Table 1, at least four of the ten enriched gene sets closely correspond to the known functions of their output genes (CDC19, UBC9, ORC6 and UTP23). However, the biological role of two output genes (YGR067C and YLR278C) remains poorly characterized and therefore, the functional correspondence with the enriched gene set could not be determined. The p-values are significantly beyond what would be expected by chance, indicating that the inferred network has substantial correspondence to known functional connections between genes. For each of the cases in the table, we computed the Šidák correction [28] and in all cases the significance level was beyond 10^-4^, conferring statistical significance to the findings.

To further validate the inferred network, we tested whether its dynamics matches the expression profiles measured after the knockout and overexpression procedures. Although the network was inferred from these experiments, the inference procedure was applied to each gene individually. Thus, it is not straightforward that the resulting network, which combines all inferred interactions, will be able to accurately mimic the expressions profiles of all genes observed in the measurements.

To test for self-consistency, we first simulated the inferred networks 10^7 ^times, starting each time from a random state. We then measured whether, given the input states, the output states are in agreement with the ones observed in the knockout and overexpression experiments. We found that each predicted output of a gene agreed with the experiments 87.4% of the time. Next, we simulated the inferred networks 10^7 ^times, starting each time from a random state and imposing one of the deletions or overexpressions performed in the measurements (randomly picked). We found that each predicted output of a gene agreed with the experiments 88.2% of the time. From the results of these tests, we conclude that the inferred network is consistent with the measurements from which it was inferred.

### Dynamics of Core Network of S. cerevisiae

We simulate the dynamics of the inferred network of *S. cerevisiae*, using the Boolean modeling strategy, and compare with the two null models. The propagation of information, quantified by *I*, was estimated as in [6], with the only difference being that we do not measure *I *from attractors, but rather from transients, since we are interested in the information propagated in the network due to perturbations and not in the long-term behaviors.

Mean transient *I *is measured as follows. We first generate 100 random initial states. For each random initial state, we generate a 'transient' time series of length 10. The probabilities used to calculate mutual information are then calculated from all 900 state transitions for each gene pair (and not by calculating the mean *I_ij _*from each individual transient and averaging over all transients, which would be mostly spurious due to their short length [6]).

It is noted that one way to assess the ability of the inferred core network to propagate information would be to start from states that the network is known to realize (such as states from the cell cycle). However, many pathways in this core network are likely to only be activated in very specific conditions (many of these are currently unknown). Thus, in order to have a broader assessment of its overall ability to propagate information, we initialize this network in random states.

The results of these measurements in the *S. cerevisiae *inferred core network as well as in the two null models are shown in Table 2. *S. cerevisiae *core network has higher mean transient *I *than both null models. We next investigate what features in the topology and/or transfer functions cause this. Table 2 also shows several structural features of these networks, namely the mean values of *K*, p-bias, *S*, *C_p_*, *L*, and the number of topologically isolated clusters of genes.

**Table 2:** Features of the topology of S. cerevisiae and the null models.

Network	⟨*K*⟩	⟨*p bias*⟩	*S*	*C_p_*	⟨*L*⟩	No. clusters	*I*
*S. cerevisiae*	5.6	0.41	0.85	0.29	4.26	11	0.014 ± 0.0003
Rand-Beta	5.6	0.41	1.24 ± 0.06	0.12 ± 0.005	3.52 ± 0.02	1.5	0.006 ± 0.001
Rand-p-bias	5.6	0.41	2.71 ± 0.1	0.29 ± 0.005	3.54 ± 0.02	1.5	0.001 ± 0.00002

"Rand-Beta" networks have the same distribution of p-bias and mean *K *but differ in *C_p _*from *S. cerevisiae*. "Rand-p-bias" networks have equal mean p-bias, *K *and *C_p _*as *S. cerevisiae *but random p-bias distribution. "No. clusters" is the number of topological clusters of nodes.

From Table 2, we first note that *S. cerevisiae *core network has a *C_p _*that is much higher than the Rand-Beta null model, where connections are randomly placed. Previous studies shown that increasing *C_p _*tends to strongly enhance *I *[21], thus explaining why *S. cerevisiae *core network exhibits much higher values of *I *than the Rand-Beta model.

In this regard, it is noted that while the *L *of *S. cerevisiae *is not significantly higher than the *L *of the Rand-Beta networks, the two networks are structurally very different. *S. cerevisiae *has a topology with "small-world" features [20] and several independent clusters (11) while the Rand-Beta networks only have, on average, 1.5 independent gene clusters.

Due to this striking difference, we tested whether the measured value of mean *I *correlates with the number of clusters. For that, we generated randomized networks with the same number of topological clusters, same mean *K *and same mean *p *- *bias *as the core network. We found no measurable difference in the values of *I *between networks with 1 to 12 clusters.

We now address the question regarding the p-bias distribution of the core network of *S. cerevisiae*, namely, its effects on information propagation. We compare *S. cerevisiae *core network with the Rand-p-bias model, which has the same *K *and *C_p _*as the *S. cerevisiae *core network. From Table 2, *S. cerevisiae *core network has a much higher *I *than the Rand-p-bias model networks.

This is explained as follows. While this beta-like p-bias distribution causes many inputs to have minor roles in determining the output state, it allows the *S. cerevisiae *inferred core network, which has a mean *K *of 5.6, to have a mean sensitivity of only 0.85, which is surprisingly close to 1, corresponding to networks that are near critical. Critical RBNs are known to maximize *I *[6]. The null model Rand-p-bias on the other hand has *S *equal to 2.71, which is deep within the chaotic regime, and thus is expected to have low *I *[6], which it does. As for the Rand-Beta model, while its *S *is also close to 1, its low *C_p _*does not allow it to have *I *as high as *S. cerevisiae*.

In short, the *S. cerevisiae *core GRN has high *C_p _*and a "beta-like" p-bias distribution that allows its sensitivity to be close to 1, despite the high connectivity. Both these features enhance *I *[21].

## Conclusions

Previous works [6,21] have hypothesized that GRNs have evolved towards maximizing temporal pairwise mutual information between the genes' expression levels, as a means to increase their degree of coordination by increasing the amount of information propagation between them. From global gene expression measurements following gene deletion and overexpression, we inferred the topology and logic of a core gene network of *S. cerevisiae*, and then simulated its dynamics using the Boolean network modeling strategy. The study of the input-output distribution showed that more genes have a very high number of inputs than expected by chance given the mean *K*, and that these genes have transfer function with p-bias close to 1 or 0. We hypothesize that these genes are preferentially regulated by a few of its TFs (under rich medium conditions), the others only being relevant in their absence or in adverse conditions. This agrees with the fact that only a small fraction of single TF deletion mutants in *S. cerevisiae *are lethal [27].

Another possible, mutually compatible explanation is that the "minor TFs" have overlapping functions. Possible approaches to investigate this include performing similar deletion experiments under conditions closer to those found in the wild, or examining multiple deletion mutants for lethal phenotypes, for genes whose single delation is non-lethal.

Contrary to what would be expected if the network was randomly wired, the inferred core network has a very high generalized clustering coefficient. This is known to enhance the ability of networks to propagate information [21]. However, another interpretation is possible for the high *C_p_*. Namely, the GRN may have evolved a high *C_p _*because it needs many clusters of small number of genes to perform specific functions that require a high degree of coordination.

Finally, we found that although the average p-bias of the transfer functions is almost unbiased, the p-bias distribution resembles a beta-like distribution with high variance, far from what is expected by chance. Because of this, although with a very high connectivity, the core network is near critical, which is known to enhance information propagation [6].

We do not know what is the cause for the high variance of the p-bias distribution. It may be merely a consequence of the inability of genes to realize complex transfer functions. In that scenario, it would be more of a hinderance in its capacity to transfer information, rather than an advantage.

The high mean connectivity and near to 0.5 mean p-bias would, however, cause the network to be "chaotic" if the distribution of p-bias was not beta-like with high variance, allowing the sensitivity to be approximately 1. Because of this, we hypothesize that the shape of p-bias distribution may have evolved to allow the core GRN of *S. cerevisiae *to be near the critical regime, consistent with the hypothesis that critical GRNs are naturally favored. The critical regime is the dynamical regime for which *I *is maximized [6].

Relevantly, in [29], it was found that critical RBNs, in comparison with ordered and chaotic ones, are those that best predict the measured distribution of genes whose activities are altered in several hundred knockout mutants of *S. cerevisiae*, supporting our finding that the core network appears to be near critical. Studies on other GRNs using different methods to assess criticality [8,30,31] have found them to be near critical as well.

We further found that the core network has a high *C_p_*. Since both features enhance information propagation within the core GRN, it may be that the maximization of propagation of information within GRNs is a general principle by which natural selection shapes the large scale topology and logic of GRNs. It is of relevance to state that while we compared the dynamics of the inferred core network with null-model networks with a random topology, we do not imply that the GRN of ancestors of *S. cerevisiae *had a more "random topology" than the present GRN of *S. cerevisiae*. From our results we can only conclude that the present core GRN of *S. cerevisiae *is able to propagate information throughout its nodes far more efficiently than standard random topologies, due to its "far from random" values of *C_p_*, *K*, and p-bias. We hypothesize that these features have been subject to selection and that, as a consequence, the present core GRN of *S. cerevisiae *is likely to be more efficient in propagating information throughout its nodes than its ancestors. Nevertheless, we cannot rule out the possibility that the present values of these "global topological" parameters result from a variety of different and independent evolutionary steps, acting at a small topological scale, which indirectly, also lead to an overall more efficient information propagation throughout the GRN.

Finally, we note that our findings are likely to rely, to some extent, on the choice of modeling strategy of GRN used (the "Boolean" approach). It will be of great interest to investigate the findings here reported using more realistic modeling strategies such as the delayed stochastic modeling strategy [4,32], shown to match measurements of gene expression at the single RNA and protein level [33]. For this to be possible, methods for quantification of information, noise, and sensitivity from stochastic temporal expression levels of RNA and protein, as well as the state of promoter (free for transcribing, bound by TFs, etc) need further development.

## Authors' contributions

The work presented here was carried out in collaboration between all authors. ASR conceived the study. All authors contributed in designing the methods, analyzing the data, interpreting the results and writing the paper. All authors have read and approved the final manuscript.

## Supplementary Material

Additional file 1 **Yeast perturbation experiments**. Each column of this tab-separated table contains the expression levels of all probe sets for one knockout experiment. The column title WT/{gene name} gives the name of the gene that was knocked out. Expression levels are given as the log base 10 of the ratio between the probe set's expression in the knockout and the wild type expression. Click here for file

Additional file 2 **Input matrix of the inferred core network**. This file contains the topology of the inferred core network. Each line contains the name of the output gene followed by a tab-separated list of input genes. Click here for file
